# Treatment of Lead Colic

**Published:** 1846-10

**Authors:** 


4. *Treatment of Lead Colic.*—During the three years that I was with M. Gendrin, I saw a vast number of cases of lead colic; we had, indeed, nearly always two or three men thus affected in our wards, sent from the carbonate of lead manufactory at Clichy. All of these cases were treated with sulphuric acid, and I do not recollect having seen one in which the disease proved refractory to the treatment adopted,—a case or two of confirmed chronic paralysis excepted. The duration of the treatment, as far as I can collect from my notes, was about three days in slight cases, and six or seven in severe ones. The sulphuric acid was given, largely diluted with water, (forty-four drops to a pint of water,) two or three pints being administered in the twenty-four hours. The amount of pure strong acid taken in that time was, therefore, from one drachm and a half to two drachms. Sometimes the sulphuric lemonade, as it was familiarly called, was vomited as soon as ingested. Still, when this was the case, the patient was made to persevere in its use, and the stomach soon became accustomed to the acid, and retained it. When it was retained, the abdominal pains generally began to diminish after the first, second, or third day, the constipation soon giving way naturally, after they had become less intense. In all these instances, not a grain of any kind of medicine was given besides the sulphuric acid, nor even was an enema used, the sulphuric acid being the only medicinal agent resorted to, if we except baths.

At the commencement of the treatment, a sulphur bath was given to the patient, the result of which was, that the sulphur combining with the particles of lead that were on the skin, formed a black sulphuret. The amount of lead which is thus discovered to encrust, as it were, the skin of those who have worked at preparations of lead, is nearly incredible. I have seen men go into the sulphur baths quite white, and come out nearly as black as negroes. The lead lying on the skin having been thus made visible to the naked eye, the patients were supplied with a hard brush and half a pound of soft soap, and made to scrub themselves daily in a warm bath, until all the black sulphuret had been brushed off. The sulphur bath was then repeated, the sulphuret of lead brought out, brushed off and the process renewed until it no longer rendered visible any trace of lead.

This precaution is indispensable with all who labor under saturnine disease, if we wish to ensure patients against relapse. Whilst at the hospitals of La Pitié and Saint Louis, I have repeatedly had patients under my care with lead colic, who had been discharged as cured from other hospitals a few weeks previously. The sulphur bath, which exhibited a thick coating of lead on the skin, explained at once the cause of the relapse. Indeed, the presence of this coating of lead on the surface of the body is, no doubt, the principle cause of the relapses which are mentioned by authors as occurring so often in these diseases. The lead which thus lies on the surface of the body is gradually absorbed, and, at last, poisoning having again taken place, all the symptoms to which it gives rise are manifested. No patient who has suffered, and been treated for lead colic, can be considered safe unless he has gone through the ordeal of a sulphur bath, with a perfectly white skin. One of the great advantages of repeating the sulphur bath during the treatment is, that the patients, whom it is easy to convince of the importance of getting rid of the metallic poison, when they see it plainly on their bodies, rub with real good will.

The mode in which the acid acts in neutralizing the poisonous effects of the lead is easy to explain. It combines, no doubt, with the lead in the tissues, and forms with it an insoluble sulphate or sulphuret, which is consequently inert, and is gradually eliminated from the economy. This is the interpretation adopted by M. Gendrin, and it appears rational enough.—Mr. Bennet in *London Lancet*, in *New York Journal of Medicine*.

